# Main Alkaloids of *Peganum harmala* L. and Their Different Effects on Dicot and Monocot Crops

**DOI:** 10.3390/molecules18032623

**Published:** 2013-02-27

**Authors:** Hua Shao, Xiaoli Huang, Yuanming Zhang, Chi Zhang

**Affiliations:** 1 Key Laboratory of Biogeography and Bioresource in Arid Land, Xinjiang Institute of Ecology and Geography, Chinese Academy of Sciences, Urumqi 830011, China; E-Mails: shaohuaconnie@yahoo.com (H.S.); huangxl0430@163.com (X.H.); zhangym@ms.xjb.ac.cn (Y.Z.); 2 State Key Laboratory of Desert and Oasis Ecology, Xinjiang Institute of Ecology and Geography, Chinese Academy of Sciences, Urumqi 830011, China

**Keywords:** phytotoxin, allelopathic, alkaloids, *Peganum harmala* L.

## Abstract

Alkaloids with allelopathic activity are not as well-known as other allelochemicals. Our study revealed that total alkaloids from seeds of the medicinal plant *Peganum harmala* L. possessed significant growth inhibitory effect on four treated plants, with dicot plants (lettuce and amaranth) being more sensitive than the tested monocot plants (wheat and ryegrass). Further investigation led to the isolation of harmaline and harmine as the main active ingredients in the total alkaloids of *P. harmala* seeds. Harmaline exerted potent inhibitory effects on seedling growth of treated plants, especially dicots, inhibiting root elongation of lettuce and amaranth by 31% and 47% at a very low concentration (5 µg/mL), whereas harmine exhibited much weaker non-selective inhibitory effect on the plants. Considering the high yield and poor utilization of *P. harmala* in China, we anticipate that this plant could be exploited as an alternative weed management tool in the future.

## 1. Introduction

*Peganum* species (family Nitrariaceae) are mainly distributed in Africa, the Middle East, central Asia, South America, Mexico, and southern USA [1,2,3]. Three species,* i.e.*, *P. harmala* L., *P. nigellastrum* Bunge and *P. multisectum* (Maxim.) Bobr. are found to grow in northwestern China, generally in arid and semi-arid regions, including Xinjiang Province, where our study site was located [3]. Among them, seeds and whole plant of *P. harmala* have a long history of use as a folk medicine in Turkey, Iran and China to treat coughs, rheumatism, hypertension, diabetes and asthma [1,4]. Phytochemical studies of *P. harmala* led to the isolation of different types of chemical ingredients such as alkaloids, steroids, flavonoids, anthraquinones, amino acids, and polysaccharides from its seeds, leaves, flowers, stems and roots [5,6,7]. Among these compounds, the alkaloids, mostly β-carbolines such as harmine, harmaline, harmalol, harmol and tetrahydroharmine, were found to be the main substances responsible for the antimicrobial, antidepressant, antinociceptive, analgesic, antitumor and vasorelaxant activities of* P. harmala* [1,8,9,10,11,12]. 

Like many other medicinal plants, *P. harmala* has been speculated to possess allelopathic properties, which presumably facilitates its dominance in its habitats and its invasive nature in southern USA [2,13]. It is suggested that allelopathy, which refers to any direct and indirect harmful or beneficial effect by one plant on another through the production of chemical compounds that are released into the surrounding environment, might influence species distribution and abundance within plant communities, and contribute to the invasion success of many exotic plants [14,15,16,17]. However, allelopathy is a notoriously difficult mechanism to demonstrate because it is hard to elucidate how putative allelochemicals might influence the community after being released into the soil or the air [18]. Still, determination of phytotoxic substances in a certain plant is usually a necessary step to evaluate whether allelopathy exists, and the dependence of allelopathic effect occurring upon release of certain compounds into the environment [19]. These compounds are usually biosynthesized in the plants as secondary metabolites. Besides functioning as an ecological factor regulating plant community composition and dynamics, plants with allelopathic traits can also be utilized either directly in weed control, or their active allelochemicals can be developed into environmentally friendly herbicides [20,21].

Previously, *Peganum* species have been reported to exhibit inhibitory effect on neighboring plants’ growth. Liu *et al.* [22,23] found that both aqueous and ethanol extracts of *P. multisectum* (Maxim.) Bobr. greatly affected seedling growth of ryegrass pea, as well as the activities of SOD, CAT and POD. Khan *et al.* [24] found that a methanol extract of *P. harmala* decreased seed germination of radish; Sodaeizadeh *et al.* [13,25] reported that aqueous extract and plant residues of *P. harmala* were toxic to treated plants, with the leaves being more toxic than stems and roots, and phenolic acids were proposed as the responsible phytotoxins. In those studies, phenolic acids were identified as the potential allelochemical candidates mainly because they were the most commonly occurring compounds with allelochemical properties according to the literature [13,26], but it is highly possible that there are other phytotoxins that contribute to the plant growth inhibitory properties of *P. harmala*. In a preliminary experiment, we found that 0.05 g/mL aqueous extract of whole *P. harmala* plant greatly suppressed growth of wheat and lettuce seedling (data not shown), indicating the presence of active phytotoxins in this plant; we thus conducted the following study to evaluate the allelopathic potential of different plant parts (leaves, stems, roots and seeds) of *P. harmala*, and to isolate and identify toxic phytochemical constituents from this plant, which might function as potential allelochemicals and possibly being utilized as cost effective natural herbicides in the future.

## 2. Results and Discussion

### 2.1. Phytotoxic Assays of Different Plant Parts of P. harmala

Ethanol extracts of leaf, stem, root and seed all exerted very strong inhibitory activity on seedling growth of wheat and lettuce (Table 1). Lettuce, a dicot plant, seemed to be more sensitive; its root length was reduced to 9%, 8%, 4% and 4% of control by 0.05 g/mL ethanol extracts of leaf, stem, root and seed, respectively, whereas root length was 26%, 16%, 17%, and 10% of control for wheat, a monocot plant. Given the fact that seeds possessed the strongest phytotoxic activity, they were chosen for further investigation.

**Table 1:** Phytotoxic effects of ethanol extracts of different plant parts of *P. harmala* at 0.05 g/mL.

	Wheat		Lettuce	
	Root length (cm)	Shoot length (cm)	Root length (cm)	Shoot length (cm)
Control	6.01 ± 0.45 ^a^	2.12 ± 0.10 ^a^	3.55 ± 0.08 ^a^	1.38 ± 0.05 ^a^
Leaf	1.54 ± 0.06 ^b^	1.11 ± 0.02 ^bc^	0.31 ± 0.01 ^b^	0.53 ± 0.04 ^bc^
Stem	0.96 ± 0.31 ^b^	1.55 ± 0.03 ^b^	0.28 ± 0.06 ^bc^	0.87 ± 0.23 ^b^
Root	1.01 ± 0.25 ^b^	0.99 ± 0.29 ^c^	0.15 ± 0.01 ^c^	0.45 ± 0.01 ^cd^
Seed	0.61 ± 0.09 ^b^	0.78 ± 0.03 ^c^	0.14 ± 0.01 ^c^	0.13 ± 0.01 ^d^

Means within a column followed by the same letter are not different at *p* < 0.05 level according to Fisher’s LSD test. Each value is the mean of three replicates ± SE.

### 2.2. Isolation and Identification of Two Toxic Alkaloids from Seeds of P. Harmala

**Figure 1:**
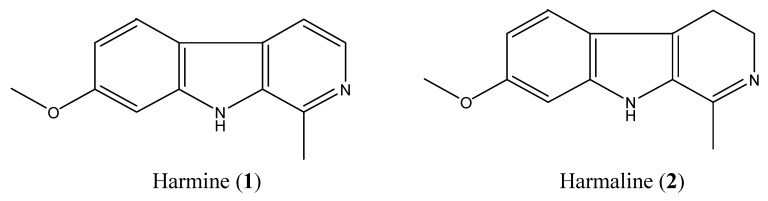
Chemical structures of harmine and harmaline.

Column chromatography and preparative TLC of total alkaloids of seeds of *P. harmala* led to the isolation of compound **1** (harmine, 1,150 mg) and compound **2** (harmaline, 140 mg; Figure 1), whose structures were identified by comparing their spectral data with published literature [4,27]. 

### 2.3. High-Performance Liquid Chromotography (HPLC) Analysis of Harmine and Harmaline

HPLC analysis indicated that the amount of harmine and harmaline varied greatly in different plant parts of the *P. harmala* samples collected from our study site. Previous studies showed that seeds and roots of *P. harmala* contained the highest levels of alkaloids, with low levels in stems and leaves, and absence in flowers [9]. Our results revealed that harmaline was only abundant in seeds (2.87%), and harmine was abundant in both seeds (2.02%) and roots (0.69%). Stems contain low amounts of harmine (0.017%) but no harmaline, and leaves had very low amounts of harmaline (0.0006%) and harmine (0.0008%). The high amount of alkaloids in seeds and roots might explain the significant inhibitory activities of their ethanol extracts; however, total contents of both alkaloids in stems and leaves of *P. harmala* were very low, indicating that the toxicity of their ethanol extracts could be attributable to other chemicals too, for instance, phenolic acids [13].

### 2.4. Phytotoxic Effects of Total Alkaloids, Harmine and Harmaline

Among the total alkaloids, harmine and harmaline, harmaline exhibited the most potent inhibitory effect on seedling growth of the tested plants, whereas total alkaloids showed relatively moderate activity, and harmine had the least inhibitory effect, all in a dose-dependent manner. Lettuce and amaranth were more sensitive to total alkaloids and harmaline, compared with the monocots wheat and ryegrass; on the other hand, the phytotoxicity of harmine did not distinguish between dicots and monocots. In comparison with the herbicide glyphosate, total alkaloids and harmine exerted weaker toxicity on receiving plants, whereas harmaline showed stronger growth inhibitory effect on the dicot plants (lettuce and amaranth), and comparatively toxic effect on wheat and ryegrass, two monocot plants (Table 2).

Root elongation of lettuce and amaranth was significantly inhibited by 30% and 43% when treated with 20 µg/mL total alkaloids; in comparison, root growth of wheat and ryegrass was not significantly affected by total alkaloids at such concentrations. When the concentration of total alkaloids increased to 100 µg/mL, root growth of two dicot plants, *i.e.*, lettuce and amaranth, was reduced by 76% and 86%, and only by 40% and 46% for wheat and ryegrass. At the highest concentration (500 µg/mL), root length of wheat, ryegrass, lettuce and amaranth was suppressed by 70%, 82%, 89% and 93%, respectively, with two dicots consistently being more sensitive than the monocots. 

Compared to total alkaloids, harmaline exhibited much stronger inhibitory effect on four treated plants, especially on dicot plants. At a very low concentration (5 µg/mL), root elongation of lettuce and amaranth was significantly affected by 31% and 47%, respectively. When the concentration reached 20 µg/mL, roots of lettuce and amaranth seedlings turned brownish, indicating the occurrence of tissue damage, which caused seedling death a few days later. In comparison, tissue damage of roots was triggered by 100 µg/mL harmaline for ryegrass, and 500 µg/mL for wheat. Seedling growth of wheat was least influenced by harmaline, which only caused 62% reduction on root growth at 100 µg/mL, but 87%, 89% and 89% root reduction on ryegrass, lettuce and amaranth, respectively. At the highest concentration (500 µg/mL), harmaline nearly completely killed seedlings of ryegrass, lettuce and amaranth, and root length of wheat was still 17% of control. On the other hand, harmine showed weaker phytotoxicity on tested plants compared to total alkaloids and harmaline. Root elongation of all receiver plants was inhibited by less than 15% when treated with harmine at 20 µg/mL; even when harmine was applied at the highest concentration (500 µg/mL), root length of wheat, ryegrass and lettuce was still 42% , 56%, and 47% of control.

**Table 2:** Phytotoxic effects of total alkaloids, harmine, harmaline and glyphosate on receiver plants at 1~500 μg/mL.

	Concentration	Amaranth		Lettuce		Wheat		Ryegrass	
	(µg/mL)	Root length	Shoot length	Root length	Shoot length	Root length	Shoot length	Root length	Shoot length
Total alkaloids	0	3.49 ± 0.16 ^a^	2.60 ± 0.09 ^a^	4.71 ± 0.18 ^a^	2.23 ± 0.07 ^a^	9.04 ± 0.28 ^a^	3.34 ± 0.15 ^a^	3.80± 0.19 ^a^	3.03 ± 0.15 ^a^
	1	3.12 ± 0.16 ^b^	1.98 ± 0.10 ^b^	4.61 ± 0.21 ^a^	1.76 ± 0.06 ^b^	8.37 ± 0.24 ^ab^	3.42 ± 0.16 ^a^	3.85 ± 0.20 ^a^	3.04 ± 0.15 ^a^
	5	3.10 ± 0.18 ^b^	1.97 ± 0.10 ^b^	4.58 ± 0.17 ^a^	1.80 ± 0.06 ^b^	8.24 ± 0.35 ^b^	3.19 ± 0.15 ^ab^	4.05 ± 0.26 ^a^	2.70 ± 0.16 ^ab^
	20	2.00 ± 0.15 ^c^	1.94 ± 0.10 ^b^	3.29 ± 0.13 ^b^	1.54 ± 0.05 ^c^	7.67 ± 0.28 ^b^	2.99 ± 0.15 ^abc^	3.95 ± 0.20 ^a^	3.03± 0.13 ^a^
	100	0.48 ± 0.03 ^d^	0.84± 0.06 ^c^	1.14 ± 0.06 ^c^	0.93 ± 0.05 ^d^	5.44 ± 0.28 ^c^	2.88 ± 0.13 ^bc^	2.07 ± 0.14 ^b^	2.31 ± 0.15 ^b^
	500	0.26± 0.01 ^d^	0.43 ± 0.03 ^d^	0.50 ± 0.04 ^d^	0.52 ± 0.03 ^e^	2.70± 0.19 ^d^	2.58 ± 0.12 ^c^	0.67 ± 0.10 ^c^	2.64 ± 0.21 ^ab^
Harmine	0	3.49± 0.16 ^a^	2.60± 0.09 ^a^	4.71 ± 0.18 ^a^	2.23 ± 0.07 ^a^	9.04 ± 0.28 ^a^	3.34 ± 0.15 ^a^	3.80± 0.19 ^ab^	3.03 ± 0.15 ^ab^
	1	3.67 ± 0.19 ^a^	2.36 ± 0.16 ^ab^	4.95 ± 0.18 ^a^	2.10 ± 0.06 ^ab^	9.21 ± 0.28 ^a^	3.53± 0.15 ^a^	4.30 ± 0.27 ^ab^	3.10 ± 0.14 ^ab^
	5	3.76 ± 0.28 ^a^	2.27 ± 0.09 ^b^	4.87 ± 0.22 ^a^	2.10 ± 0.06 ^ab^	9.11 ± 0.33 ^a^	3.22 ± 0.14 ^ab^	4.19 ± 0.19 ^a^	3.19 ± 0.17 ^a^
	20	2.95 ± 0.19 ^b^	2.23 ± 0.11 ^b^	4.09± 0.18 ^b^	1.94 ± 0.07 ^b^	8.44 ± 0.34 ^a^	3.16± 0.13 ^ab^	3.33 ± 0.23 ^b^	2.60 ± 0.14 ^c^
	100	1.19 ± 0.08 ^c^	1.74 ± 0.09 ^c^	3.41± 0.18 ^c^	1.43 ± 0.04 ^c^	6.16 ± 0.38 ^b^	2.84 ± 0.16 ^b^	3.29 ± 0.17 ^b^	2.38± 0.14 ^c^
	500	0.17 ± 0.05 ^d^	1.30 ± 0.08 ^d^	2.20 ± 0.24 ^d^	1.20± 0.05 ^d^	3.83 ± 0.19 ^c^	2.70 ± 0.11 ^bc^	2.11 ± 0.15 ^c^	2.72± 0.12 ^bc^
Harmaline	0	3.49± 0.16 ^a^	2.60± 0.09 ^a^	4.71 ± 0.18 ^a^	2.23 ± 0.07 ^a^	9.04 ± 0.28 ^a^	3.34 ± 0.15 ^a^	3.80± 0.19 ^a^	3.03 ± 0.15^a^
	1	1.89 ± 0.18 ^b^	2.16 ± 0.11 ^b^	4.18 ± 0.23 ^b^	1.87 ± 0.06 ^b^	8.31 ± 0.36 ^a^	3.13 ± 0.12 ^ab^	3.93 ± 0.23 ^a^	3.26± 0.14 ^a^
	5	1.84 ± 0.17 ^b^	2.19 ± 0.14 ^b^	3.26 ± 0.12 ^c^	1.85 ± 0.07 ^b^	7.30 ± 0.26 ^b^	3.07 ± 0.14 ^ab^	3.78 ± 0.19 ^a^	3.28 ± 0.16 ^a^
	20	0.94 ± 0.08 ^c^	1.40 ± 0.08 ^c^	1.78 ± 0.11 ^d^	1.46 ± 0.03 ^c^	6.45 ± 0.25 ^c^	2.82 ± 0.14 ^bc^	2.22 ± 0.14 ^b^	3.14 ± 0.14 ^a^
	100	0.40 ± 0.02 ^d^	0.29 ± 0.03 ^d^	0.54 ± 0.16 ^e^	0.48 ± 0.07 ^d^	3.43 ± 0.20 ^d^	2.54 ± 0.15 ^c^	0.50± 0.07 ^c^	2.58 ± 0.13 ^b^
	500	0.22 ± 0.02 ^d^	0.20 ± 0.02 ^d^	0.20 ± 0.01 ^e^	0.00 ± 0.00 ^e^	1.58 ± 0.19 ^e^	1.88 ± 0.16 ^d^	0.02 ± 0.01 ^d^	0.56 ± 0.17 ^c^
Glyphosate	0	3.49± 0.16 ^a^	2.60± 0.09 ^a^	4.71 ± 0.18 ^a^	2.23 ± 0.07 ^a^	9.04 ± 0.28 ^a^	3.34 ± 0.15 ^bc^	3.80± 0.19 ^b^	3.03 ± 0.15 ^c^
	1	3.81 ± 0.17 ^a^	2.11 ± 0.06 ^b^	4.29 ± 0.27 ^b^	2.24± 0.06 ^a^	10.13 ± 0.34 ^b^	4.44 ± 0.34 ^a^	4.63 ± 0.20 ^a^	4.21 ± 0.12 ^a^
	5	3.47 ± 0.19 ^a^	2.19 ± 0.07 ^b^	4.02 ± 0.18 ^b^	2.17 ± 0.06 ^ab^	7.99± 0.31 ^c^	3.81 ± 0.20 ^b^	2.32 ± 0.13 ^c^	3.52 ± 0.13 ^b^
	20	2.57 ± 0.11 ^b^	2.50 ± 0.09 ^a^	1.63 ± 0.06 ^c^	1.91 ± 0.09 ^c^	4.34 ± 0.21 ^d^	3.10 ± 0.20 ^c^	1.33 ± 0.09 ^d^	3.25 ± 0.11 ^bc^
	100	1.46 ± 0.08 ^c^	2.48 ± 0.06 ^a^	1.10 ± 0.06 ^d^	2.01 ± 0.07 ^bc^	2.72 ± 0.15 ^e^	1.84 ± 0.13 ^d^	0.87 ± 0.05 ^e^	2.63± 0.11 ^d^
	500	0.95 ± 0.06 ^d^	2.61 ± 0.09 ^a^	0.70 ± 0.04 ^e^	1.46 ± 0.04 ^d^	1.45 ± 0.19 ^f^	0.82 ± 0.12 ^e^	0.10 ± 0.03 ^f^	0.19 ± 0.07 ^e^

Means within a column followed by the same letter are not different at *p* < 0.05 level according to Fisher’s LSD test. Each value is the mean of three replicates ± SE.

Amaranth, whose root elongation was only 5% of control when cultivated in 500 µg/mL harmine solution, was the most sensitive plant. Under *in vitro* bioassay conditions, both aqueous and ethanol extracts of *P. harmala* exhibited significant inhibitory effects on treated plants. It is evident that this medicinal plant can produce phytochemical compounds with plant growth inhibitory activities. Total alkaloids from seeds of *P. harmala* were found to possess strong plant growth inhibitory activity, and harmaline and harmine were identified as the major responsible compounds. Compared to other isolated allelochemicals, the toxicity of harmaline is relatively strong, and harmine is rather weak [16,19,28,29,30]. 

It is believed that almost all plant species can produce chemicals that are toxic to one species or another; therefore, it is insufficient to declare the occurance of allelopathy of a certain plant species simply because of the presence of phytotoxins [31]. In fact, phytotoxins will not function as active allelochemicals unless they can be released into the environment, and persist in toxic forms in the medium (soil/air/water) at allelopathic levels for a certain period of time [32,33,34]. Therefore, it needs to be demonstrated that these alkaloids can be released into the soil matrix, possibly via leaching, litter decomposition and root exudation; meanwhile, like other allelochemicals, the fate of these alkaloids depends greatly on the environment. Once they enter the soil, these chemicals are exposed to various physicochemical and biological processes, which might trigger degradations or chemical reactions that lead to the production of novel compounds with different biological activities [35]. On the other hand, besides allelopathy, *P. harmala* also possess other biological characteristics that might contribute to its dominance and invasiveness. For example, *P. harmala* is extremely drought tolerant; its deep taproot is characterized with 2 or 3 rings of anomalous vascular bundles surrounding the central cylinder, which is considered to be an important adaption to dry conditions [36]; it is unpalatable to animals [37]; *etc.* Taken together, we believe that the ecological success of *P. harmala* is attributable to the combination of various biological properties, possibly including its inherent allelopathic traits, thus, further investigation is needed to demonstrate the possible involvement of allelopathy.

Synthetic herbicides are widely used in weed management, however they are usually toxic and may cause environmental problems. Moreover, overuse of certain chemical herbicides has created the problem of development of herbicide-resistant weeds. By 1998, 216 herbicide-resistant weed biotypes had been recorded in 45 countries, and the number of new herbicide-resistant weeds continues to increase, at an average of nine new cases per year worldwide [38]. Under such circumstances, developing environment-friendly new herbicides by utilizing natural products as lead compounds seems to be one approach to help solve these problems. There have been successful examples of using natural products, including allelochemicals, as sources to develop commercial herbicides [39]; for instance, mesotrione, a synthesized analogue of leptospermone that is produced by the “bottle brush” plant *Callistemon citrinus* [40], and cinmethylin, a derivative of 1,4-cineole that is a natural phytotoxin found in the essential oils of a number of plants [41]. More importantly, natural phytotoxins were found to act on a large number of unexploited herbicide target sites, which can be used to deal with the rapid evolving resistance to synthetic herbicides [42]. Besides utilizing allelochemicals as herbicides, plants with allelopathic properties can also be applied in integrated weed management, for instance, they can be used as cover crops, intercrops, green manure, and so on [21,43]. In a greenhouse experiment conducted by Sozaeizadeh *et al.* [25], plant residues of *P. harmala* were found to significantly suppress seedling growth of two wild weeds, indicating the possibility of utilizing this plant in weed control. Considering the high yield and poor utilization of *P. harmala*, we anticipate that this plant could be exploited as an alternative weed management tool in the future.

## 3. Experimental

### 3.1. Instrumentation

IR spectra were measured on a Perkin-Elmer 783 spectrometer with KBr disks. UV spectra were recorded on a Perkin Elmer Lambda 25 UV/VIS spectrometer. ^1^H- and ^13^C-NMR spectra were recorded on a Varian Unity Inova instrument (at 400/100 MHz, respectively). EIMS were measured on a Micromass Platform EI-200 GC/MS instrument at 70 eV by direct insertion probe. HPLC was performed with a model 1100 series Agilent Technologies HPLC system. Chromatographic separation was achieved on a Phenomenex C18 Column (4.6 × 250 mm, 5 µm).

### 3.2. Materials and Reagents

Whole plants of *P. harmala* (including leaf, stem and root) were collected in Urumqi, Xinjiang province in June, 2011. Seeds were collected at the same location in August, 2011. Plant materials were air dried in our laboratory in the shade at room temperature (22~25 °C, controlled by air conditioning) for two weeks before use. Harmine and harmaline (98% purity) were purchased from BioBioPha Co., Ltd (Kunming, China). Glyphosate (Roundup^®^) was produced by Monsanto Co., St. Louis, MO, USA.

### 3.3. Phytotoxic Effects of Ethanol Extracts of Different Plant Parts

The air dried plant materials of *P. harmala* were separated into leaves, stems and roots. Five g of each part and seeds were ground into powder and macerated in 95% ethanol (100 mL) for 24 h to give 0.05 g/mL ethanol extracts. A dicot plant, lettuce (*Lectuca sativa* L.), and a monocot plant, wheat (*Triticum aestivum *L.), were used as test plants for this assay. Crop plants were used in the bioassay because of their high seed germination rate and uniformity of germination and seedling growth. Test seeds were surface sterilized with 0.5% HgCl_2_ before use. Three mL of ethanol extract of each plant part were then added to each Petri dish (9 cm diameter) lined with Whatman No. 1 filter paper. After complete evaporation of ethanol, distilled water (3 mL) was added to each Petri dish followed by addition of 10 seeds. Petri dishes were sealed with Parafilm to prevent water loss and stored in the dark at 25 °C. Seedlings were allowed to grow for 4 days before shoot and root lengths were measured. Three replicates were made for all phytotoxic bioassays (in total 30 seedlings were measured).

### 3.4. Extraction and Isolation of Phytotoxins

**Figure 2:**
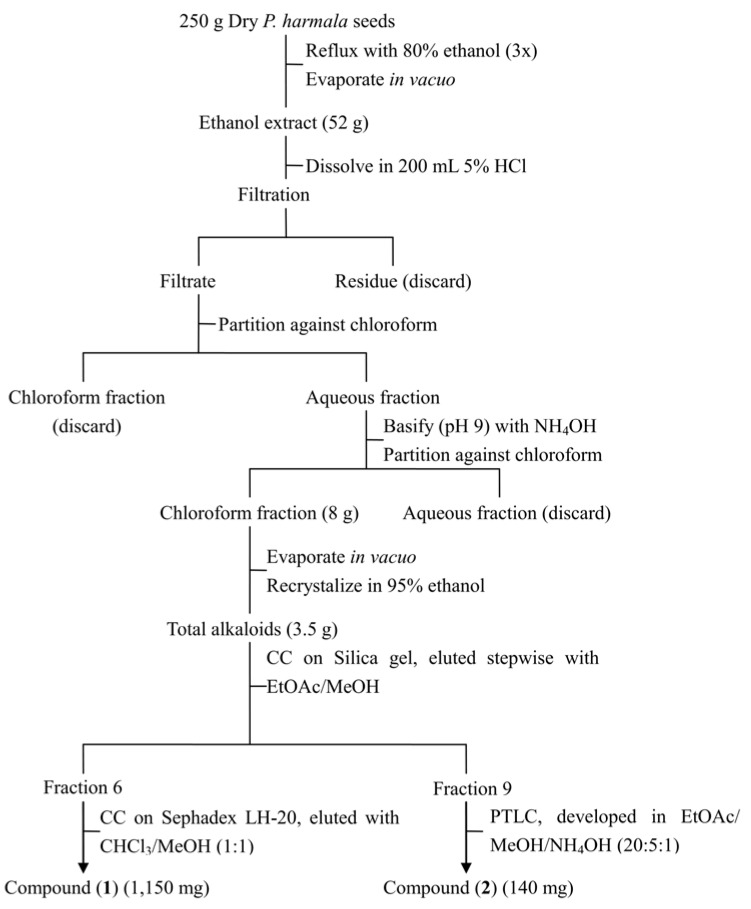
Protocol for isolation of harmaline and harmine.

Seeds of *P. harmala* exhibited the strongest inhibitory activity, therefore they were chosen for further study. Three hundred grams of seeds were ground into powder and extracted three times by refluxing with 80% ethanol (500 mL). The ethanol extract was concentrated under reduced pressure to yield 52 g of a dark red residue which was subsequently dissolved in HCl (5%, 200 mL) and filtered. The filtrate was then partitioned three times with chloroform (200 mL); the chloroform extracts were combined and dried under reduced pressure to give 250 mg of substance, which did not show any major spots on a TLC plate and was then discarded. The aqueous acid layer was made alkaline to pH 9 with NH_4_OH and extracted four times with chloroform (200 mL) to yield 8 g (dry weight) of chloroform extract. The chloroform extract was then recrystallized in ethanol to give 3.5 g of yellowish crude crystals of total alkaloids. The crystals were further separated on a silica gel column eluted with a step gradient elution (EtOAc/MeOH at 1:0, 98:2, 96:4, 9:1, 8:2, 7:3, 6:4, 1:1, 0:1). Seventeen fractions were collected based on TLC profiles, which were subsequently tested for their phytotoxicity. The most toxic fractions, fractions 6 and 9, were selected for further purification. Fraction 6 was then subjected to a Sephadex LH-20 column chromatography (2 × 150 cm), which led to the isolation of harmine (1,150 mg); fraction 9 was purified by preparative TLC to give 140 mg harmaline (Figure 2). Structures of both compounds were identified by comparing their spectral data with published literature [4,27].

### 3.5. HPLC Analysis of Harmine and Harmaline

Seeds and roots (0.2 g), and stems and leaves (6 g) were weighed into flasks separately and ground into powder. Powder of each plant part was then mixed with methanol (20 mL for seeds and roots or 200 mL for stems and leaves). The flasks were then sonicated in a bath sonicator at room temperature for 30 min. The methanol extracts were filtered separately, and the same volume of methanol was added to each flask and the sonication procedure repeated twice. Filtered methanol extracts were pooled, concentrated *in vacuo*, and dissolved to final volumes of 10 mL to obtain 0.02 g/mL (seeds and roots) and 0.6 g/mL (stems and leaves) methanol extracts. Three random samples were prepared for each plant part for the HPLC analysis. The HPLC injection volume was 10 µL, and the methanol extracts of different plant parts were eluted with 20% acetonitrile in 0.1 M ammonium acetate over 25 min at a flow rate of 1 mL·min^−1^. The column effluent was monitored at 330 nm [27]. Harmine and harmaline (98% purity, purchased from BioBioPha Co., Ltd., Kunming, China) were used as standards to determine the amount of harmine and harmaline in each sample (samples were diluted when necessary).

### 3.6. Phytotoxic Effects of Total Alkaloids, Purified Harmine and Harmaline

Phytotoxic activity of the total alkaloids, purified harmine and harmaline obtained from the above purification procedure was assessed by conducting bioassays against two dicot plants, amaranth and lettuce, and two monocots, wheat and ryegrass, at 1, 5, 20, 100 and 500 μg/mL concentrations, using similar procedures for testing the phytotoxic effect of ethanol extracts of *P. harmala*, with ethanol being the initial solvent for the alkaloids. Purity of purified harmine (98%) and harmaline (96%) used in the bioassays was first examined by TLC and then determined by ^1^H-NMR and HPLC analysis. Seedlings were allowed to grow for 4 (for lettuce and wheat) or 5 (for ryegrass and amaranth) days before shoot and root length were measured. 

### 3.7. Statistical Analyses

The significance of the phytotoxic effects of extracts of *P. harmala*, total alkaloids, harmine, harmaline and glyphosate on seedling growth of test species was first examined by ANOVA (*p* < 0.05) and then analyzed using Fisher’s LSD test at *p* < 0.05 level.

## 4. Conclusions

Under *in vitro* bioassay conditions, both aqueous and ethanol extracts of *P. harmala* exhibited significant inhibitory effects on receiver plants. It is evident that this medicinal plant can produce phytochemical compounds with plant growth inhibitory activities. Total alkaloids of seeds of *P. harmala* exerted significant inhibitory activity on treated plants, with the two tested dicots being more sensitive. Further study led to the isolation and identification of two alkaloids, harmaline and harmine, as the main active phytotoxins in the total alkaloids. Harmaline possessed strong inhibitory activity on treated plants, with dicots being more sensitive, whereas harmine showed non-selective and relatively weak activity. HPLC analysis revealed that harmaline was abundant only in seeds of *P. harmala*, while harmine was abundant in both seeds and roost, indicating that stems and leaves of *P. harmala* must contain other phytochemical constituents that contribute to the toxicity of their ethanol and aqueous extracts.
